# Prefrontal Cortex Activation Upon a Demanding Virtual Hand-Controlled Task: A New Frontier for Neuroergonomics

**DOI:** 10.3389/fnhum.2016.00053

**Published:** 2016-02-16

**Authors:** Marika Carrieri, Andrea Petracca, Stefania Lancia, Sara Basso Moro, Sabrina Brigadoi, Matteo Spezialetti, Marco Ferrari, Giuseppe Placidi, Valentina Quaresima

**Affiliations:** ^1^Department of Life, Health and Environmental Sciences, University of L’AquilaL’Aquila, Italy; ^2^Department of Developmental Psychology, University of PadovaPadova, Italy; ^3^Department of Physical and Chemical Sciences, University of L’AquilaL’Aquila, Italy

**Keywords:** functional near-infrared spectroscopy, neuroergonomics, hand-controlled task, LEAP motion controller, virtual reality, remote control, brain activation

## Abstract

Functional near-infrared spectroscopy (fNIRS) is a non-invasive vascular-based functional neuroimaging technology that can assess, simultaneously from multiple cortical areas, concentration changes in oxygenated-deoxygenated hemoglobin at the level of the cortical microcirculation blood vessels. fNIRS, with its high degree of ecological validity and its very limited requirement of physical constraints to subjects, could represent a valid tool for monitoring cortical responses in the research field of neuroergonomics. In virtual reality (VR) real situations can be replicated with greater control than those obtainable in the real world. Therefore, VR is the ideal setting where studies about neuroergonomics applications can be performed. The aim of the present study was to investigate, by a 20-channel fNIRS system, the dorsolateral/ventrolateral prefrontal cortex (DLPFC/VLPFC) in subjects while performing a demanding VR hand-controlled task (HCT). Considering the complexity of the HCT, its execution should require the attentional resources allocation and the integration of different executive functions. The HCT simulates the interaction with a real, remotely-driven, system operating in a critical environment. The hand movements were captured by a high spatial and temporal resolution 3-dimensional (3D) hand-sensing device, the LEAP motion controller, a gesture-based control interface that could be used in VR for tele-operated applications. Fifteen University students were asked to guide, with their right hand/forearm, a virtual ball (VB) over a virtual route (VROU) reproducing a 42 m narrow road including some critical points. The subjects tried to travel as long as possible without making VB fall. The distance traveled by the guided VB was 70.2 ± 37.2 m. The less skilled subjects failed several times in guiding the VB over the VROU. Nevertheless, a bilateral VLPFC activation, in response to the HCT execution, was observed in all the subjects. No correlation was found between the distance traveled by the guided VB and the corresponding cortical activation. These results confirm the suitability of fNIRS technology to objectively evaluate cortical hemodynamic changes occurring in VR environments. Future studies could give a contribution to a better understanding of the cognitive mechanisms underlying human performance either in expert or non-expert operators during the simulation of different demanding/fatiguing activities.

## Introduction

The term neuroergonomics was first introduced in 1997 for depicting an interdisciplinary area of research which involves the intersection of two disciplines: neuroscience and ergonomics (Parasuraman and Rizzo, 2007). The studies in this field, previously carried out using either mobile or immobile neuroimaging techniques, have been nicely reviewed by Mehta and Parasuraman (2013). Virtual reality (VR), a computer-based technology that allows the creation of multisensory simulated environments in which users can interact and receive real-time feedbacks on their performance, was claimed by Kearney et al. (2007) to be highly relevant for neuroergonomics. This because VR can replicate, with a greater control than that applicable in the real world, a wide range of conditions that are impractical or impossible to observe in the real situations; then allowing behavioral and neurophysiological observations of the mind and brain at work. Given its peculiarity, VR is also effectively used by human operators to accomplish their work in dangerous environments, thus avoiding any physical risk. For instance, applying gesture-based control interfaces, VR is usually employed for tele-operated applications such as driving robots, rovers and other devices remotely, with the operators at a certain distance from them (Chen et al., 2015; Liu and Zhang, 2015; Wei et al., 2015). The tele-operated systems are very expensive, unique neither replicable nor quickly replaceable, and from their proper use depends the success or failure of long-planned, critical, costly and challenging operations. Taking into account the high degree of responsibility inherent to operators’ duties, their considerable physical/cognitive work should be evaluated objectively by neuroimaging techniques in the framework of neuroergonomics (for review, see Gramann et al., 2011, 2014; Mehta and Parasuraman, 2013).

Although the most widely used immobile functional neuroimaging modality has been undoubtedly represented by functional magnetic resonance imaging (fMRI), the development of portable and wearable neuroimaging devices, comprising electroencephalography (EEG) and functional near infrared spectroscopy (fNIRS; for review, see Mehta and Parasuraman, 2013; Gramann et al., 2014; Scholkmann et al., 2014) has considerably facilitated the approach of neuroergonomics. fNIRS, with its high degree of ecological validity and its very limited requirement of physical constraints to subjects, represents a valuable tool for monitoring cortical responses in the research fields of neuroergonomics (for reviews, see Ayaz et al., 2013; Derosière et al., 2013). Furthermore, compared to fMRI, fNIRS is silent, allowing to avoid any bias in the results due to difficulties in focusing on the task because of the high level of noise. Briefly, fNIRS is a non-invasive vascular-based functional neuroimaging technology which assesses, simultaneously from multiple measurement sites, concentration changes in oxygenated-deoxygenated hemoglobin (O_2_Hb/HHb, respectively) at the level of the cortical microcirculation blood vessels (Scholkmann et al., 2014). O_2_Hb/HHb, indeed, interact differently with near infrared light, so that both physiological indexes can be recovered from the measured signal. This is a further advantage of fNIRS over fMRI, the latter being able to recover only a single physiological index, namely the blood-oxygen level dependent (BOLD) signal. When a specific brain region is activated, cerebral blood flow increases in a temporally and spatially coordinated manner through a complex sequence of coordinated events, tightly linked to changes in neural activity (i.e., neurovascular coupling). The coupling between the neuronal activity and the cerebral blood flow is fundamental to brain function. fNIRS relies exactly on this coupling to reveal the activated cortical region by measuring the associated cortical blood oxygenation changes (i.e., the increase in O_2_Hb and the decrease in HHb).

Since 1993, fNIRS has been employed for evaluating the spatiotemporal characteristics of the cortical activation during different motor tasks related to upper and/or lower limb exercise (for a review, see Leff et al., 2011). In most previous fNIRS studies, the activation of the sensory-motor cortex and the PFC has been widely investigated in different tasks of the lower limb like walking (Koenraadt et al., 2014; Mirelman et al., 2014), stepping (Huppert et al., 2013), precision stepping (Koenraadt et al., 2014), etc. Several fNIRS studies have also investigated the PFC activation during hand tasks such as: finger movement (Wriessnegger et al., 2012), passive finger movement (Chang et al., 2014), isometric grasping/grasping (Mandrick et al., 2013), handgrip exercise (Derosière et al., 2014), learning a hand motor skill (Hatakenaka et al., 2007), etc.

Since 2000, fNIRS has been also employed in real-world activities realized in VR environment for evaluating the PFC activation during the simulation of different hand-related demanding/fatiguing activities, like airplane piloting (Ayaz et al., 2012a; Durantin et al., 2014; Gateau et al., 2015), car driving (Tomioka et al., 2009), grasping (Holper et al., 2010, 2012), natural orifice transluminal endoscopic surgery (James et al., 2011), etc. Interestingly, the inferior frontal gyrus (Ayaz et al., 2012a) and the dorsolateral PFC (DLPFC; Durantin et al., 2014; Gateau et al., 2015) were found activated during airplane piloting tasks. The bilateral ventrolateral PFC (VLPFC) was found activated during natural orifice transluminal endoscopic surgery when the simulation required a more difficult navigation path through an orifice (James et al., 2011).

It is well-known that the PFC, and in particular the VLPFC and DLPFC, are involved in the control of the motor actions. On one hand, it has been demonstrated that the VLPFC is involved in visuo-motor learning tasks (Yamagata et al., 2012; Hoshi, 2013). Moreover the reflexive orienting seems to be controlled by the right VLPFC (Corbetta et al., 2008), whereas the goal relevant information for the action control seems to be maintained and retrieved by the left VLPFC (Badre and Wagner, 2007; Souza et al., 2009). On the other hand, the DLPFC apparently plays a specific role in learning by trial and error (Halsband and Lange, 2006). Furthermore DLPFC, for its involvement in mediating and monitoring of actions, is considered to be the major anatomical correlate of the central executive (Baddeley, 2003; Gateau et al., 2015). Therefore, the VLPFC and the DLPFC are involved in associating visual information with motor responses (Halsband and Lange, 2006; Tanji and Hoshi, 2008).

The PFC plays not only a crucial role in single cognitive or motor tasks, but also in combined sensorimotor-cognitive task (i.e., dual-task; Gentili et al., 2013; Mandrick et al., 2013; Mirelman et al., 2014). Several fNIRS studies have reported that, in comparison to a single task, the attention-demanding dual-tasks (e.g., walking while talking, calculating while stepping, balancing a ball while walking, etc.) induced an increase of the PFC activation due to a greater cognitive load (Holtzer et al., 2011). For instance, Mandrick et al. (2013) investigated how an additional mental load (i.e., arithmetic task) during isometric grasping affects the PFC activation. The performance of the mental task was impaired when the motor task difficulty increased, suggesting that performing a dual-task requires more attentional resources than performing a single task.

In the last few years, the use of VR interfaces, driven by natural hand movements for remote control, is growing-up thanks to the development of innovative optical 3-dimensional (3D) systems for gesture recognition (Erden and Çetin, 2014). The key advantage of gesture recognition technology is that no physical contact is required between the human body and the gesture recognition device, so that the subjects can move freely. One of the most recent optical 3D sensors, based on stereo-vision, is the LEAP Motion Controller^®^ (LEAP). The LEAP is a high-resolution 3D hand-sensing device, which allows the freehand natural interaction crucial for the implementation of real-time, realistic VR systems. This low cost non-bulky device has an extremely accurate reactivity (Bachmann et al., 2014). In addition, the 3D rendering technologies, including state of the art displays and visors specifically designed for VR (Dodgson, 2013; Desai et al., 2014; Nan et al., 2014), have permitted a large development of VR techniques. In particular, their high visual/rendering fidelity and an immersive wide field of view enables the sensation of presence and the feeling being actually inside the virtual scene with the possibility to have multi-sensorial feedbacks.

The aim of the present study was to investigate non-invasively by fNIRS the PFC responses in healthy subjects while performing a complex hand-controlled task (HCT) in a VR environment. This task emulated the interaction with a real, remotely-driven, system operating into a critical environment. The hand movements were captured by the LEAP, a high spatial and temporal resolution 3D hand-sensing device. The subjects were asked to move their right hand/forearm with the purpose of guiding a virtual ball (VB) over a virtual route (VROU). The VROU can be easily and purposely designed to replicate a real track that an operator should travel to carry on a given challenging operation. The HCT-related PFC response was monitored non-invasively by a 20-channel fNIRS system. The HCT involved the control of the hand/forearm movement, and the active interaction with the virtual environment through the hand/forearm motor actions. The attentional resources allocation and the integration of different executive functions (e.g., coordination, planning, decision making, etc.) are needed in performing the HCT. Taking into account the well-known role played by the VLPFC and the DLPFC in motor action preparation and in the allocation of the attentional resources to generate goals from current situations, it was hypothesized that either VLPFC or DLPFC or both of them would be activated bilaterally in subjects while performing the HCT.

## Materials and Methods

### Participants

Fifteen University students (all males, age: 26.6 ± 2.9 years; level of education: 14.4 ± 2.1 years), without neurological or psychiatric illness and normal or corrected-to-normal vision were recruited in the study. In order to prevent any gender differences in emotional responses (Matud, 2004) and in visuo-motor abilities (Wang et al., 2015), only men were enrolled. To exclude left-handed subjects, all participants completed the Edinburgh Handedness Inventory assessing hand dominance. Following a full explanation of the protocol and its non-invasiveness, and prior to the starting of the experimental procedure, a written informed consent was obtained from each participant. All procedures were conducted in accordance with the Declaration of Helsinki and approved by the University Ethics Committee.

### Experimental Setup

#### Hand-Controlled Task (HCT)

**Figure 1:**
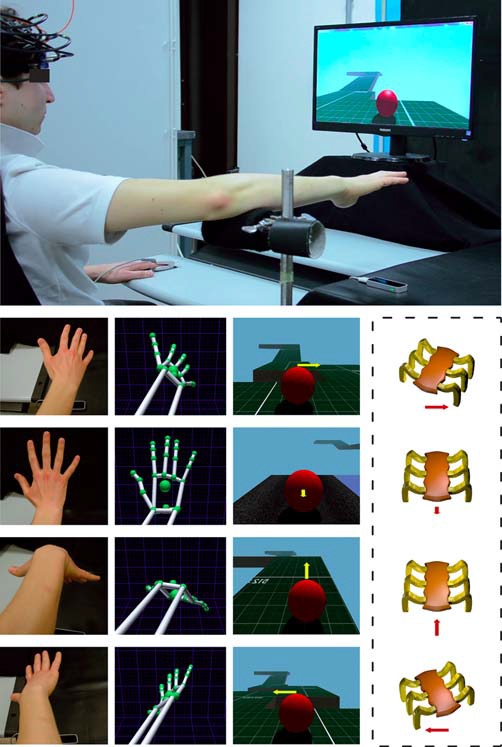
**Experimental setting for the execution of a virtual reality (VR) hand-controlled task (HCT).** The upper large image shows the subject with the functional near-infrared spectroscopy (fNIRS) probe holder while sitting in front of a PC, and the positioning of the LEAP motion controller below the operator hand. The four columns of small images show (from left to right): the real position of the subject hand/forearm (first column); the corresponding hand/forearm virtual model (second column); the influence of the commands on the virtual ball (VB) during the HCT (third column); and the corresponding effects on a real, remote, spider-like rover (fourth column, in dashed line to indicate that the presence of the rover is just supposed). The yellow arrow indicates the direction of the guided VB and the red arrow indicates the corresponding effect on the rover. The length of the arrows indicates the force amplitude impressed by the operator. Note that, during the HCT, the hand/forearm virtual model (second column) was not shown to the operator and the visualization of the arrows (third column) was disabled.

A VR HCT was implemented by integrating a LEAP Motion Controller^®^ with a real-time 3D engine. The LEAP provides both a 3D hand model and real-time hand tracking information for enabling subjects to transpose their hand movements within the virtual 3D HCT (Figure 1). The LEAP is a small (1.3 cm × 3.2 cm × 8 cm) 3D sensor which uses two internal infra-red (IR) cameras and three IR light emitting diodes to detect objects within a dome of approximately 0.22 m^3^ above it. Its spatial and temporal resolution is 1 mm and 15 ms, respectively. The LEAP, connected to a computer via a USB cable, is designed specifically to detect, in real-time, hand and finger motions and gestures, such as pinching fingers, closing hand, tapping, etc. This device, positioned under the palm center of the right hand at a distance of about 25 cm (Figure 1), was utilized to: (1) capture the movements of the hand; (2) associate hand movements to a virtual hand model; and (3) translate the movements of the virtual hand model to a set of commands in order to drive a VB within a virtual environment.

**Figure 2:**
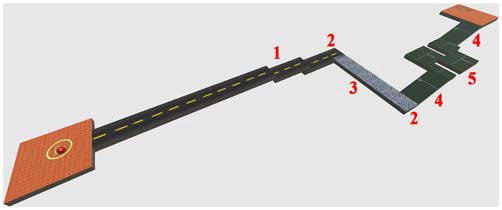
**A perspective view of the designed virtual route (VROU) which reproduces a narrow road including some critical points.** 1: stairs; 2: turns; 3: slippery part; 4: climbs; and 5: sequential turns.

In the present study, the adopted virtual environment was aimed at simulating the driving action of a spider-like robot similar to the one developed by the National Aeronautics and Space Administration (NASA). Due to its high stability, equilibrium and ability to change quickly direction, this robot has been proven to be adapted for moving in very rough environments and in environments designed for humans (to go up and down stairs). The movements of the ball, in fact, simulated fairly those ones performed by the considered robot (lateral, ahead, behind, and stop). The aim of this adopted HCT was to move the VB over a VROU of a fixed length (42 m; Figure 2), trying to travel as long as possible the distance in a fixed time without falling (2 min). Either in the case of VB falling or in the case of accomplishment of a VROU in advance, subjects were requested to restart the VROU from the beginning. To calculate the whole distance traveled by each subject over the HCT, the completed VROU and the distance traveled until the task end were considered. The VROUs with failure were not considered. The VROU adopted in this study (Figure 2) was purposely designed to reproduce a narrow road including some critical points (i.e., stairs, turns, a slippery part, and climbs). The Torque 3D Engine^1^, a cross-platform high performance real-time 3D engine, was used both for the editing and the rendering of the whole virtual 3D HCT. A controllable VB and a 3D VROU were created by using a customized version of Marble Motion, a well-known game^2^. In particular, the whole native source code was rewritten and enriched in order to fulfill the requirements of the task: (1) a time driven version of the task in which both the start and the end of the HCT were established by a fixed time interval; (2) the possibility of storing information related to the operator-task interaction process (including the number of times in which the VB was fallen out of the VROU, the position of the VB falls over the VROU, the distance traveled by the VB in the given time). The software allowed also the calculation of some dynamic parameters (followed trajectory, VB speed and acceleration). These last parameters have been considered to calculate the VB speed along the VROU, to get information about resting periods occurred during the HCT, and to evaluate the subject skills while executing the HCT. Moreover, to increase the subject’s concentration and motivation during the HCT, the whole technical items, including elements of the VROU (textures and materials), were redefined utilizing some predefined items of the 3D engine editor (Torque 3D Editor)^3^.

The subject was asked to place his right forearm on a fixed and firm support in order to allow the hand capture (Figure 1). This support ensured the maintenance of the correct position of the forearm, and consequently of the hand, during the execution of the HCT. The task started with a stationary VB placed at the beginning of the VROU. The subject had to maintain his right hand opened over the LEAP device by keeping his forearm on the support with the center of the palm perpendicular to the center of the device and with all the five fingers extended (Figure 1). At the beginning of the HCT, the subject had to guide the VB over the VROU by using four commands (Figure 1). The first command (hand flexion) made the VB to proceed forward; the second command (hand extension) made the VB to decrease the speed (up to stop the VB) and to proceed backward; the third and the fourth commands (counter clockwise and clockwise rotations of the wrist) made the VB to move toward left or right, respectively (a combined use of the hand flexion/extension movements or the rotation of the wrist made the VB to stop). These hand movements had a real-time proportional impact on the VB. More specifically, the command chosen by the subject transmitted the direction and the “force” to the VB (e.g., a low hand downward flexion corresponded to a low “force” application to the VB in the forward direction), especially when the VB speed was depending on the inclination degree of the downward flexion and the time during which the hand was maintained at the same position. Thus, the HCT was purposely designed to combine the four main commands and when the subject assumed a pose of his hand halfway between two commands, the system merged both directions and “force” amplitude. In this way, the subject had the feeling to guide the VB without restrictions or constraints.

The effective range of the LEAP tracking system is limited to roughly 60 cm due to the low near infrared light intensity. In this study, the distance between the fNIRS head probe and the LEAP was always greater than 60 cm. Therefore, the LEAP should have not interfered with the fNIRS measurements. Interestingly, several fNIRS studies in combination with other near infrared based tracking systems such as Kinect or eye trackers have been published (Kita et al., 2010; Sukal-Moulton et al., 2014; Urakawa et al., 2015). Although those systems have utilized more powerful light emitters (than the LEAP), and the light emitters have been directly pointed toward the fNIRS head probe, the potential interference with the fNIRS data was not mentioned.

#### fNIRS Instrumentation and Data Processing

A two-wavelength continuous wave 20-channel fNIRS system (Oxymon Mk III, Artinis Medical Systems, Netherlands) was utilized to map non-invasively the changes in O_2_Hb and HHb over the bilateral PFC. The details of this instrumentation have been previously reported (Basso Moro et al., 2013). The O_2_Hb/HHb data from the 20 channels were acquired at 10 Hz. The O_2_Hb/HHb concentration changes (expressed in ΔμM), obtained by using the modified Beer-Lambert law and the age-dependent differential pathlenght factor (4.99 + 0.067 × Age^0.814^) were displayed in real-time on a PC monitor. Eight optical fiber bundles (length: 3.15 m; diameter: 4.5 mm) were utilized to transport the light to the left and the right PFC (four for each hemisphere), whereas ten optical fiber bundles of the same size (five for each hemisphere) were utilized to collect the light emerging from the PFC. The illuminating and collecting bundles were assembled into a flexible probe holder, consisting of two mirror-like units (9.7 cm × 8.9 cm each) held together by three flexible junctions. In 16 out of the 20 channels the illuminator-detector distance was set at 3.5 cm, while in the remaining four channels the illuminator-detector distance was set at 1 cm (short-separation channels or SS channels). In the 16 channels, the measurement points were defined as the midpoint of the corresponding illuminator-detector pairs. The probe holder was placed over the subject head by a Velcro brand fastener in order to get a stable optical contact with the scalp (Figure 1). In particular, the two frontopolar fibers bundles, collecting the light at the bottom of the holder, were centered (according to the International 10–20 system for the EEG electrode placement) on the Fp1 and Fp2 locations for the left and right hemisphere, respectively. The pressure created by the fastener was sufficient to induce a partial transient blockage of the skin circulation during the fNIRS study. The adopted procedure would suggest that a consistent reduction of forehead skin blood flow was occurring as a result of this approach. The Montreal Neurological Institute coordinates of the optodes and the relative 16 measurement points were calculated using a probe placement method. For the details of this procedure see Basso Moro et al. (2013). The measurement points 1, 2, 3, 9, 10, 11 corresponded to the DLPFC, which includes part of the Brodmann’s Area (BA) 46; the measurement points 5, 6, 13, 14 corresponded to the frontopolar cortex, which includes part of the BA 10; and measurement points 4, 7, 8, 12, 15, 16 corresponded to the VLPFC, which includes part of the BA 45.

During the data collection procedure, the fNIRS signal quality as well as the absence of movement artifacts were verified on the PC monitor. The subject’s heart rate (HR) was monitored by a pulse oximeter (N-600, Nellcor, Puritan Bennett, St. Louis, MO, USA) with the sensor clipped to the index finger of the left hand. The Homer2 NIRS processing package^4^ was employed to analyze the data. Raw intensity data in each channel were converted into optical density changes (OD). Channels showing low intensity values were excluded from further analyses. The Wavelet motion correction method was employed to correct motion artifacts. Based on the method developed by Molavi and Dumont (2012), it sets to zero all wavelet detail coefficients exceeding a predefined threshold (iqr = 0.1). The modified Beer-Lambert law was then applied to convert the corrected OD data into concentration changes. A General Linear Model (GLM) approach (hmrDeconvHRF_DriftSS) was utilized to recover the mean hemodynamic response function (HRF) for each subject and channel. The approach, consisting in adding the SS channel signal with the highest correlation with the analyzed standard channel signal in the design matrix, was able to reduce the contribution of the mean arterial blood pressure changes in task-evoked fNIRS signal. The less restrictive set of Gaussian functions with standard deviation (SD) of 3 s and with their means separated by 2 s was chosen as temporal basis functions (ranging between −20 before and 210 s after the starting of the HCT; Gagnon et al., 2011).

#### Experimental Design

A familiarization/training phase was carried out 3 days before the study. The subjects were informed about the procedures and familiarized with both the experimental setting and the HCT. During this phase, the fNIRS probe holder was placed over the head of the subjects who were trained to stay as firm as possible to avoid movement artifacts in fNIRS measurement during the HCT execution. After evaluating the joint mobility of their hand and wrist, the subjects were requested to pay attention to the presented four commands to be used for guiding properly the VB (hand flexion, hand extension, counter clockwise and clockwise rotations of the wrist). Later, the subjects were asked to place their right hand opened over the LEAP in order to verify the correctness of the estimated hand virtual model. Once this phase was completed, the subjects were asked to guide a VB over a VROU. In order to avoid a potential learning effect, a different VROU was used in this training phase. For each subject, the training phase was considered completed when he demonstrated his ability to guide properly the VB. After 3 days, at the same time of the training phase, the subjects participated in the study. A monetary reward was not given.

This study was carried out in a quiet and dimly lit room. The subject was asked to sit on a comfortable high-backed chair in front of a 17″ PC monitor, and to keep his forearm on the firm support with the right hand opened over the LEAP (see “The Hand-Controlled Task” Section; Figure 1). The HCT protocol lasted 6 min. Specifically, the protocol started with a 3 min baseline, during which subjects were asked to relax (observing a white fixation cross presented on a black screen) in order to get stable fNIRS signals. Then, a stationary VB came into view on the PC monitor, and a visual instruction informed the subjects that the 2 min HCT was starting. During the HCT, the subject had to guide the VB over the VROU through the four hand movements (see “The Hand-Controlled Task” Section). When the subject failed in guiding the VB over the VROU or the subject completed the VROU in less than 2 min, the VB was repositioned at the VROU starting point, and the route restarted. At the end of the HCT execution, there was a recovery period (1 min), in which the subject was requested to relax while observing a white fixation cross presented on a black screen. In order to evaluate the potential “state anxiety” provoked by HCT, all the subjects completed the 20-items of the State Trait Anxiety Inventory Form Y-1 (STAI) before and after the protocol.

### Data Analysis and Statistics

The integral values of the O_2_Hb/HHb (_INT_O_2_Hb/HHb) changes of the HRFs were calculated from the beginning (at 0 s) until the end of the HCT (at 120 s), for each measurement point and subject, and were used as metric for the following statistical analysis. The mean values of the HR changes (analyzed as percentage of control) were calculated from the beginning (at 0 s) until the end of the HCT (at 120 s). Both the _INT_O_2_Hb/HHb changes and the HR mean values were corrected for the baseline periods, calculated over the last 20 s before the starting of the HCT. The median of the values of the distance traveled by the guided VB was calculated to subdivide the subjects in two groups: best performers (above the median) and worst performers (below the median). The subject of the median value was not included in any group. Student’s *t*-test was conducted in order to evaluate the presence of any difference, in terms of the distance traveled by the guided VB between the worst and best performers.

All data were examined for normality and sphericity using Shapiro–Wilk and Mauchly’s Sphericity tests, respectively. Each level of the independent variables followed a normal or approximately normal distribution in all the dependent variables (O_2_Hb, HHb and HR), permitting the use of parametric statistical analyses. When the sphericity was not assumed, the Greenhouse-Geisser correction was utilized.

In order to investigate the PFC activation in response to HCT, the two-way analysis of variance (ANOVA) was applied to _INT_O_2_Hb/HHb changes. The ANOVA included two factors: measurement point (16 levels) and cortical hemodynamic response (CHR; i.e., corrected task period vs. zero; 2 levels). To control for multiple significance tests, the Fisher’s least significant difference adjustment was applied. A series of one-way ANOVAs was performed for the HCT in order to evaluate the influence of the CHR (2 levels) on the _INT_O_2_Hb/HHb changes. In particular, the one-way series of ANOVAs were performed only for the _INT_O_2_Hb/HHb changes related to the measurement points 7, 8, 15 and 16, chosen as descriptive measurement points of the hemodynamic response. For the HCT, the Pearson’s correlation coefficient was calculated in order to evaluate the relation between the distance traveled by the guided VB and the _INT_O_2_Hb/HHb changes in the 7, 8, 15 and 16 measurement points. A one-way ANOVA was performed for the HCT in order to evaluate the influence of the CHR (2 levels) on the HR changes. The Pearson’s correlation coefficient was also calculated for evaluating the relation between the distance traveled by the guided VB and the HR. Student’s *t*-tests were conducted in order to evaluate the presence of any difference in: (1) the anxiety state before and after the protocol; (2) _INT_O_2_Hb/HHb changes in the 7, 8, 15 and 16 measurement points between the worst performers and best performers; and (3) the distance traveled by the guided VB of worst and best performers.

All statistical analyses were conducted with SPSS 20.0 (SPSS Inc., Chicago, IL, USA). Data were expressed as mean ± *SD*. The criterion for significance was *p* < 0.05.

## Results

**Figure 3:**
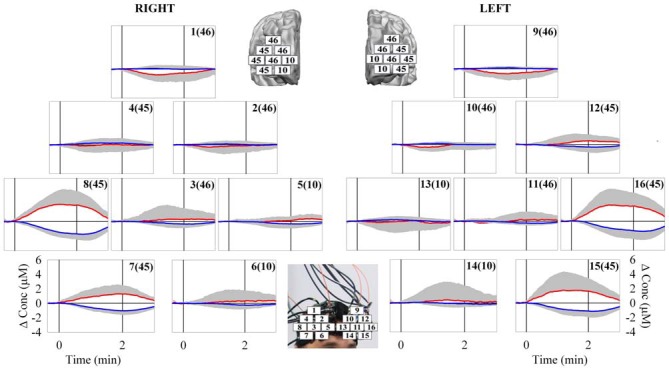
**Grand average of O_2_Hb (red line) and HHb (blue line) changes observed over the bilateral PFC [16 measurement points of the right (from 1–8) and left (from 9–16) hemisphere] in response to the VR HCT.** The corresponding numbers in the brackets refer to the associated Brodmann’s Area (BA). The vertical solid lines limit the duration of the task execution. The major cortical activation was observed in the bilateral VLPFC (measurement points 7, 8, 15, and 16). *N* = 15; means ± *SD*.

The behavioral data analysis revealed the following main results. There was no significant difference (*t* = 0.64, *p* = 0.53) in the anxiety state before (28.8 ± 6.4) and after the protocol (28.9 ± 5.8). The distance traveled by the guided VB in the 15 subjects was: 21, 26, 36, 43, 51, 53, 56, 58, 61, 83, 88, 96, 101, 132, and 148 m, respectively. The median value was 58 m. The mean distance was 70.2 ± 37.2 m. The less skilled subjects failed several times in guiding the VB over the VROU. Therefore, the distance traveled by their guided VB was shorter. Indeed, the distance traveled by the guided VB was significantly different (*t* = −4.89, *p* < 0.001) between the worst performers (40.9 ± 13.7 m) and best performers (101.3 ± 29.7 m). The fNIRS data evidenced a heterogeneous O_2_Hb/HHb response over the mapped cortical area in the subjects while performing the HCT (Figure 3). In particular, since the beginning of the HCT, a progressive O_2_Hb increase and a concomitant progressive HHb decrease were observed in the measurement points 7, 8, 15 and 16, corresponding to the VLPFC, which includes part of the BA 45. About 15 s after the end of the HCT, a gradual return of O_2_Hb/HHb to the corresponding baseline values, was observed. This delay is reasonable considering that the cerebral blood flow increase lasts over the period of the HCT. The statistical analysis revealed the following main results. The two-way ANOVA analysis, carried out on the _INT_O_2_Hb changes, revealed a significant main effect of: (1) the measurement point (*F*_(2.67,37.47)_ = 9.52, *p* < 0.001), and (2) the measurement point * CHR interaction (*F*_(2.67,37.47)_ = 9.52, *p* < 0.001). The two-way ANOVA analysis, carried out on the _INT_HHb changes, revealed a significant main effect of the: (1) measurement point (*F*_(2.83,39.67)_ = 15.70, *p* < 0.001); (2) CHR (*F*_(1.00,14.00)_ = 13.83, *p* = 0.002); and (3) measurement point * CHR interaction (*F*_(2.83,39.67)_ = 15.70, *p* < 0.001). The two-way ANOVAs, carried out on the _INT_O_2_Hb/HHb changes, revealed the main significant differences between the measurement points 7, 8, 15, 16 and all the others (*p*s < 0.05). The series of one-way ANOVAs, carried out on the _INT_O_2_Hb changes of the measurement points 7, 8, 15, and 16, revealed a significant activation in all the measurement points (*F*_(1,14)_ = 5.32, *p* = 0.037; *F*_(1,14)_ = 23.79, *p* < 0.001; *F*_(1,4)_ = 7.33, *p* = 0.017; *F*_(1,14)_ = 16.89, *p* = 0.001).The series of one-way ANOVAs carried out on the _INT_HHb changes of the measurement points 7, 8, 15, and 16 revealed a significant cortical activation in all the measurement points (*F*_(1,14)_ = 41.61, *p* < 0.001; *F*_(1,14)_ = 58.57, *p* < 0.001; *F*_(1,14)_ = 19.12, *p* = 0.001; *F*_(1,14)_ = 26.23, *p* < 0.001). In the HCT, no correlation was found between the distance traveled by the guided VB and the corresponding _INT_O_2_Hb/HHb changes (*p*s > 0.05). During the HCT, no differences (*p*s > 0.05) were found in the _INT_O_2_Hb/HHb changes in the 7, 8, 15 and 16 measurement points between the worst performers and best performers. The one-way ANOVA analysis for the HR mean values revealed a significant main effect of the CHR (*F*_(2.88,40.40)_ = 8.06, *p* < 0.001). However, the mean values of the HR changes during the execution of the HCT increased only of about 15% with respect to the mean value of the baseline. No correlation was found between the HR and the distance traveled by the guided VB.

## Discussion

In this feasibility study, the bilateral PFC was investigated by a multi-channel fNIRS system while subjects performed a demanding VR HCT, a remotely-driven operation simulated by a high-resolution and low-cost 3D hand-sensing device. The observed involvement of the bilateral VLPFC supports the formulated hypothesis.

The results of the present study have indicated a consistent bilateral VLPFC activation (measured as O_2_Hb increase and a concomitant HHb decrease) in response to the execution of the VR HCT (Figure 3). It has been reported that VLPFC is involved in associating visual information with motor responses (Tanji and Hoshi, 2008). In fact, the execution of the adopted HCT requires the combination of the contextual visual information in order to coordinate the hand/forearm movements for guiding the VB over the VROU. Although the tested subjects had the same age and level of education (University students), their skills in performing the HCT were different. Indeed, the distance traveled by the VB was heterogeneous: the distance traveled by the VB guided by the best performer was about seven times longer than the distance traveled by the VB guided by the worst performer. These results clearly confirm that the designed VROU (Figure 2) was really demanding. The diverse skills of the subjects could not be attributable to emotional factors, because no difference between anxiety state before and after the HCT, and no correlation between the distance traveled by the guided VB and the HR of the subjects were found. However, a bilateral VLPFC activation was observed indiscriminately in all the tested subjects, including the ones who never completed at least one VROU. Therefore, the present fNIRS data did not provide the possibility to discriminate the subjects according to their performance. This could be partly explained by the fact that other cortical areas were not investigated in this study and that subcortical areas and/or cortical-subcortical network are supposed to be responsible of the differences between the best and the worst performers. As well known, PFC is involved in the executive functions (e.g., attention, coordination, planning, decision making, etc.), the same required to perform the HCT regardless of the performer’s skills. The combined use of a fNIRS-EEG system has evidenced a greater involvement of the deeper structures (e.g., hippocampus) in the “good performers group” compared with the “bad performers group” while executing a spatial navigation task (Kober et al., 2013). Moreover, it has been reported that a complex sensorimotor-cognitive task, such as the adopted HCT, would require the involvement of different cortical-subcortical networks including: PFC, spinal cord, brainstem, cerebellum, basal ganglia, and motor cortex (Takakusaki, 2008). This suggests that the degree of the cognitive demand (measured by fNIRS), required for executing the HCT, is not associated with the subjects performance (measured as the distance traveled by the guided VB). This in part confirms the results of other VR fNIRS studies in which a dissociation between the mental work demanded to execute a complex task and the performance output was observed (Ayaz et al., 2012a; Boyer et al., 2015). In the present study, this dissociation could be also explained by the fact that tested subjects had only a short familiarization/training phase. A higher activation of the PFC was usually observed in non-expert subjects while executing a novel VR task compared to expert (Ayaz et al., 2012b). The non-expertise requires the employment of more attentional resources to perform a novel task. On the contrary, the expertise implies an increase of automaticity and does not require the same high level of attention and control (Ayaz et al., 2012b). The VLPFC activation, observed in the present study in non-expert subjects while performing the novel VR HCT, suggests that some of the well-known executive functions (e.g., attention, coordination, planning, decision making, etc.) are required in the learning phase. Then, it could be supposed that the amplitude of the observed VLPFC activation would become lower or even disappear in subjects very familiar with the VR HCT.

Several studies evidenced the advantages of using fNIRS technology for investigating non-invasively cortical responses in subjects while performing different VR tasks; the most representative studies are listed in Table 1. The common relevant finding is represented by the activation of different regions of the frontal cortex and the PFC. For example, the involvement of the medial PFC (mPFC) and the frontopolar cortex (Ayaz et al., 2012b) and the inferior frontal gyrus (Harrison et al., 2014) was observed in subjects while executing air traffic control tasks; an activation of the overall frontal cortex was observed in subjects while performing a train piloting task (Kojima et al., 2005). Very recently, the usefulness of fNIRS as a tool to conduct driving research has been nicely reviewed (Liu et al., 2015). For example, an activation of the right PFC (Tomioka et al., 2009) and an activation of the overall PFC (Tsunashima and Yanagisawa, 2009) were found in subjects while executing different simulated car driving tasks. However, in all of the above reported studies, no activation of the VLPFC, and in particular of the BA 45, was found.

**Table 1:** **Selected fNIRS studies about the effects of VR tasks on different cortical areas**.

VR Tasks	Subjects (patients)	Age (years)	D	Ch	Cortical areas	Main findings	Reference
Air traffic control	24	24−55	D1	16	FP, PFC	Medial FP/PFC activated	Ayaz et al. (2012b)
Air traffic control	12	NA	D1	16	PFC	PFC activated	Harrison et al. (2014)
Airplane piloting	9	36 ± 4	D7	1	FC	FC differently activated by task difficulty	Takeuchi (2000)
Airplane piloting	13	21−28	D1	16	FP, PFC	IFG differently activated by practice	Ayaz et al. (2012a)
Airplane piloting	12	25 ± 5	D1	16	PFC	Correlation DLPFC activation/ performance	Durantin et al. (2014)
Airplane piloting	19	27 ± 6	D1	16	FP, PFC	DLPFC activated bilaterally	Gateau et al. (2015)
Balancing (*swing*)	16	29 ± 5	D2	8	PFC	PFC activated bilaterally	Basso Moro et al. (2014)
Balancing (*tilt board*)	22	26 ± 4	D2	8	PFC	PFC activated bilaterally	Ferrari et al. (2014)
Balancing *(video game)*	9	18−42	D3	32	FC, MC, SC, TC	STG activated	Karim et al. (2012)
Boxing (*video game*)	20	18−40	D4	46	ATC, SMA	ATC and SMA differently activated	Kim et al. (2015)
Car driving	9	NA	D5	42	FC	FC less activated during ACC	Tsunashima and Yanagisawa (2009)
Car driving	14 (12 AD)	NA	D4	52	PFC	PFC less activated in AD	Tomioka et al. (2009)
Dancing	14	22 ± 1	D5	22	(L)PFC, (L)TC	Training-dependent PFC activation	Ono et al. (2015)
Dancing	26	NA	D6	22	(L)PFC, (L)TC	MTG activated	Noah et al. (2015)*
Grasping	23	NA	D8	4	M1, PC PMC, SMA	M1, PMC, SMA, PC activated	Holper et al. (2010)
Grasping	17	26 ± 4	D8	4	PMC, SMA	PMC/SMA differently activated by trials	Holper et al. (2012)
Lathe operation	7	24−26	D6	45	FC, MC	FC and MC activated	Hou and Watanuki (2012)
Line bisection	8	28	D9	20	OC, PC	OC and PC activated	Seraglia et al. (2011)
Line-tracking	2	NA	D8	4	PMC	PMC activated	Brand et al. (2011)
Maze	2	NA	D1	16	PFC	PFC more activated during BLK	Ayaz et al. (2011)
Maze	15 GP	24 ± 1	D4	24	(R)FC,	PC activated	Kober et al. (2013)**
	12 BP	28 ± 1			(R)PC		
Missile defense	30	18−31	D9	2	PFC	PFC activated	Boyer et al. (2015)
Shopping	6	61 ± 16	D10	16	PFC	PFC more activated in BD	Okahashi et al. (2014)
	(10 BD)	23 ± 1					
Surgery	29	32 ± 6	D4	24	PFC	Lateral PFC more activated in experts	James et al. (2011)
Surgery	20	29 ± 2	D4	24	OC, PC	OC less activated by improved performance	Leff et al. (2015)
Surgery	7	23−26	D4	24	PC	IS activated	Miura et al. (2015)
Train driving	2	NA	D5	44	FC, OC	FC widely activated in manual condition	Kojima et al. (2005)
Walking (*haptic touch on treadmill*)	7 (1 CS)	25 ± 9	D4	44	PFC, PMC, SC, SMA	PFC and SMA widely/locally activated	Sangani et al. (2015)

ACC, adaptive cruise control; AD, Alzheimer’s disease; ATC, anterior temporal cortex; BD, brain damage patients; BLK, blocked order; BP, bad performers; Ch, number of channels; CS, chronic stroke patient; D, Device; D1, Imager 1000 (fNIR Devices, USA); D2, NIRO-200 (Hamamatsu Photonics, Japan); D3, CW6 (TechEn, USA); D4: ETG-4000 (Hitachi, Japan); D5, OMM-3000 (Shimadzu, Japan); D6, FOIRE-3000 (Shimadzu, Japan); D7, NIRO-500 (Hamamatsu Photonics, Japan); D8, Wireless prototype (Zurich University, Switzerland); D9, Imagent (ISS, USA); D10, OEG-16 (Spectratech Inc., Japan); DLPFC, dorsolateral prefrontal cortex; FC, frontal cortex; FP, frontopolar; GP, good performers; IFG, inferior frontal gyrus; IS, intraparietal sulcus; (L), left; M1, primary motor cortex; MC, motor cortex; MTG, middle temporal gyrus; NA, not available; OC, occipital cortex; PC, parietal cortex; PFC, prefrontal cortex; PMC, premotor cortex; (R), right; SC, somatosensory cortex; SMA, supplemental motor area; STG, superior temporal gyrus; TC, temporal cortex; VR, virtual reality; *fNIRS-fMRI study; **combined fNIRS-EEG study.

The combined use of fNIRS-EEG would be an ideal tool for carrying out studies in the field of neuroergonomics. The pros of lightweight, high-density EEG and fNIRS recording to study natural human cognition have been previously reviewed (Gramann et al., 2011, 2014). Simultaneous fNIRS-EEG measurements offer complementary functional information about neuronal activity and hemodynamic changes in order to provide a wider perspective on different aspects of the cortical processes. To the best of our knowledge, Kober et al. (2013) have first compared neuronal responses of good and bad navigators during a VR spatial navigation task by a combined fNIRS-EEG system. The commercial fNIRS systems, utilized either by Kober et al. (2013) or in the present study, are equipped with fiber optic bundles. The disadvantage of using fiber optic bundles is that the fibers are often heavy and with a limited flexibility. Therefore, this kind of fNIRS instrumentation is not the best choice for studies in the neuroergonomics field. Since 2009, different battery operated multi-channel wearable/wireless fNIRS systems have been commercialized (Scholkmann et al., 2014). A 64-measurement point wireless fNIRS system was developed and integrated with simultaneous EEG and electrocardiography (ECG) monitoring in order to record data up to several days (Zhang et al., 2014). These most advanced versions of integrated fNIRS-EEG systems represent a suitable tool for evaluating brain activation in response to cognitive tasks executed in normal daily activities. To make fNIRS technology more suitable for neuroergonomics studies, in terms of robustness, mobility, user-friendliness and customization, a very recent and successful effort has been made to realize a dedicated fNIRS device (von Lühmann et al., 2015). Further future hardware developments will increase the production of miniaturized wireless integrated fNIRS-EEG devices and their use in neuroergonomics. It is noteworthy to mention that a 16-measurement point wireless fNIRS system has been recently coupled with transcranial direct current stimulation (tDCS) in order to investigate the effects of tDCS on spatial working memory (McKendrick et al., 2015). These authors have suggested the utility of using the combination of simultaneous tDCS and fNIRS techniques for future applications in the field of neuroergonomics: from enhanced/accelerated learning and training of complex human-machine systems to optimization of task load for improved safety and productivity. The neuroergonomics approaches can also provide sensitive and reliable assessment of mental workload in complex tasks and naturalistic work settings (Parasuraman, 2011). The mental workload has been defined as the degree of the effort to be made by the brain to meet the task demands (Young et al., 2015). Recently, Peck et al. (2014) have reviewed the use of fNIRS to measure mental workload in the real world tasks, and the different approaches for automatic detection of the workload.

The strengths and the limitations of the fNIRS technique have been previously discussed in detail (for review, see Scholkmann et al., 2014). The fNIRS equipment is transportable, completely safe and non-invasive. These advantages allow for the investigation of brain activity in natural conditions (e.g., while sitting on a chair) and during daily life activities (e.g., standing and/or walking). Therefore, with respect to other functional neuroimaging methods such as fMRI, fNIRS represents an useful tool for neuroergonomics research (for review, see Ayaz et al., 2013; Derosière et al., 2013), for studies in other fields of neuroscience such as brain-computer interface (for review, see Naseer and Hong, 2015), human-robot interaction (for review, see Canning and Scheutz, 2013), and cognitive states measurements (for review, see Strait and Scheutz, 2014). In addition, very recently the integration of fNIRS with a wearable technology, such as Google Glass, has been demonstrated (Afergan et al., 2015). For an adequate understanding of the current findings, some limitations should be pointed out: (1) this study has been conducted in a small sample of healthy young male adults subjects and the subjective cognitive load was not tested by NASA Task Load Index; (2) the duration of the adopted task and the length of the route were relatively short, hence, the effect of a longer duration of the VR HCT on the PFC hemodynamic response remains unknown; (3) this study did not imply a control session for example including/not including motor task with/without VR; (4) this study did not contemplate repeated trials on separate days in order to verify the reproducibility and the potential learning effect of the HCT; (5) the limited number of measurement points (16) made possible by the utilized fNIRS system did not allow the investigation of the supposed connectivity between the PFC and other cortical areas (e.g., premotor and motor cortices) likely involved in performing HCT; and (6) this study did not take into account the impact of the variability of the skull thickness amongst the 16 measurement points within subject and amongst subjects. This anatomical variability could be examined by acquiring structural T1-weighted MRI scans from each subject.

## Conclusion

The results of the present study confirm the promising application of fNIRS technology to objectively evaluate cortical hemodynamic changes occurring in VR environments. The ongoing development of fNIRS technology, finalized to deliver more dedicated, sophisticated and wireless devices, together with the most advanced VR solutions, could provide the best combined approach for monitoring operators training and assessing mental work. Future studies could give a contribution to a better understanding of the cognitive mechanisms underlying human performance either in expert or non-expert operators.

## Author Contributions

Study design and protocols were conceived by MC, AP, SL, SBM, MS, MF, GP and VQ. Data collection was performed by MC, AP, SL, SBM, MS, MF, GP and VQ. Data analysis was performed by MC, AP, SL, SBM, SB, MS, MF, GP and VQ. The manuscript was written by MC, AP, SL, SBM, SB, MS, MF, GP and VQ.

## Conflict of Interest Statement

The authors declare that the research was conducted in the absence of any commercial or financial relationships that could be construed as a potential conflict of interest.

## Footnotes

^1^http://www.garagegames.com/products/torque-3d

^2^http://mit.garagegames.com/MarbleMotion-1-0b.zip

^3^http://www.garagegames.com/products/torque-3d/overview/editor

^4^http://www.nmr.mgh.harvard.edu/PMI/resources/homer2/home.htm
